# Slow-binding reversible inhibitor of acetylcholinesterase with long-lasting action for prophylaxis of organophosphate poisoning

**DOI:** 10.1038/s41598-020-73822-6

**Published:** 2020-10-06

**Authors:** Oksana A. Lenina, Irina V. Zueva, Vladimir V. Zobov, Vyacheslav E. Semenov, Patrick Masson, Konstantin A. Petrov

**Affiliations:** 1 Arbuzov Institute of Organic and Physical Chemistry, Federal Research Center “Kazan Scientific Center of the Russian Academy of Sciences”, Arbuzov str., 8, Kazan, Russian Federation 420088; 2 Kazan Federal University, 18 Kremlyovskaya str, Kazan, Russia 420008

**Keywords:** Drug discovery, Toxicology, Neuroscience, Synaptic transmission

## Abstract

Organophosphorus (OP) compounds represent a serious health hazard worldwide. The dominant mechanism of their action results from covalent inhibition of acetylcholinesterase (AChE). Standard therapy of acute OP poisoning is partially effective. However, prophylactic administration of reversible or pseudo-irreversible AChE inhibitors before OP exposure increases the efficiency of standard therapy. The purpose of the study was to test the duration of the protective effect of a slow-binding reversible AChE inhibitor (C547) in a mouse model against acute exposure to paraoxon (POX). It was shown that the rate of inhibition of AChE by POX in vitro after pre-inhibition with C547 was several times lower than without C547. Ex vivo pre-incubation of mouse diaphragm with C547 significantly prevented the POX-induced muscle weakness. Then it was shown that pre-treatment of mice with C547 at the dose of 0.01 mg/kg significantly increased survival after poisoning by 2xLD_50_ POX. The duration of the pre-treatment was effective up to 96 h, whereas currently used drug for pre-exposure treatment, pyridostigmine at a dose of 0.15 mg/kg was effective less than 24 h. Thus, long-lasting slow-binding reversible AChE inhibitors can be considered as new potential drugs to increase the duration of pre-exposure treatment of OP poisoning.

## Introduction

Organophosphorus (OP) compounds as pesticides and banned chemical warfare agents are serious health concerns worldwide^1^. It was estimated that OP-based pesticides are responsible for 200 000 fatal cases of intoxications every year^2^. Moreover, despite an international treaty that prohibits the use of chemical warfare agents, several OP warfare agents have recently been used in terrorist and criminal acts^3,4^.

OP compounds irreversibly block acetylcholinesterase (AChE) and butyrylcholinesterase (BChE), enzymes that terminate the synaptic action of the neurotransmitter acetylcholine (ACh). Inhibition results from phosphylation of the catalytic serine of these enzymes. This leads to a major cholinergic syndrome. Respiratory failure during acute cholinergic crisis is the main cause of death^5^.

Standard emergency therapy of OP poisoning consists of atropine, which prevents hyperactivation of muscarinic receptors, benzodiazepines to control seizures and oximes, to reactivate cholinesterases (ChE) by dephosphylating the active site serine^2^.

If OP exposure is expected, prophylactic countermeasures may be taken to mitigate poisoning and increase the efficiency of standard therapy. It was shown that the efficacy of standard therapy of acute nerve agent poisoning can be improved if reversible or pseudo-irreversible ChE inhibitors are given before exposure^6–14^. Administered as a pre-treatment, these non‐OP ChE inhibitors bind transiently to AChE, thereby protect its active site from irreversible inhibition by OPs^14,15^. It is important to emphasize that for a better protection, mitigating pre-exposure treatment must be followed by atropine, oxime, anticonvulsants, and bioscavengers if available, in case of OP poisoning symptoms^16^. However, it was reported that protection against OPs can be achieved by using reversible ChE inhibitors without the need of post-exposure treatment^17^. An alternative approach for pre‐exposure treatment is the sole administration of different types of bioscavengers (e.g. human BChE, evolved paraoxonase-1) that neutralize OP molecules in the bloodstream before reaching cholinergic synapses^16^.

At the moment, only the pseudo-irreversible ChE carbamylating agent pyridostigmine bromide is FDA-approved for military use as a pre‐exposure treatment when OPs poisoning is anticipated^9^. This ChE inhibitor was initially developed to rescue synaptic transmission at the neuromuscular junctions (NMJ) in *myasthenia gravis*. It poorly penetrates blood–brain barrier^18,19^ and has currently been used for decades for symptomatic therapy of different types of muscle weakness^20^.

Since the pharmacological effect persists only while ChE is carbamylated, symptomatic muscle weakness therapy as well as pre‐exposure treatment of OPs poisoning require multiple administrations of drug for sustained long-term levels of ChE inhibition. Recommended prophylactic dosing of pyridostigmine for humans is 30 mg every 8 h, during the period of risk exposure to OP^16,21^. Thus, for a given dose, the efficiency of pre‐treatment is highly dependent on pharmacokinetics (PK) and pharmacodynamics (PD) of the used ChE inhibitor.

In this context, it is important to note that architecture of NMJs, containing high density of AChE (5000 AChE molecules/µm^2^)^22^ in a small crowded space (synaptic cleft), determines a sub-compartment at the origin of micro PK/PD mechanisms. The structure of NMJ determines high target occupancy of ligands and increases residence time of ligands on targets due possible re-binding. As a consequence, ligand-binding kinetics controls the duration of drug action in micro-anatomical compartments. Therefore, potent slow-binding ligands with slow rate of dissociation from targets, re-binding and slow exit rate from micro-anatomical compartments, such as NMJ, display long-lasting action^23^.

Concepts and methodology for analysis of micro PK/PD mechanisms and drug discovery have been developed in the past decade^23–33^. However, at the moment, very few slow-binding inhibitors of AChE have been used for their pharmacological properties and only one of them has been analyzed in terms of a micro PK/PD mechanism^23,34^.

We previously described selective mammalian AChE inhibitors based on 1,3-bis[ω-(substituted benzylethylamino)alkyl]-6-methyluracils^23,35–40^. Recent PK/PD study of C547 (1,3-bis[5-(diethyl-*o*-nitrobenzylammonium)pentyl]-6-methyluracil dibromide), the most specific 6-methyluracil derivative for AChE, revealed micro-PD mechanisms taking place in NMJ^23^. The binding kinetics of C547 to AChE is characterized by a long residence time on target (τ = 20 min)^39^ and a slow diffusion rate (exit rate) out of NMJ^23^. This makes possible re-binding of C547 to AChE and therefore, slow elimination from NMJ. As the result of binding thermodynamics, kinetic selectivity and micro-anatomical features of NMJ, C547 has a long-lasting action on skeletal muscles, higher than 72 h in rat model of *myasthenia gravis*^23^.

The present study was designed to test the hypothesis that acute toxicity of the OP paraoxon (POX) can be decreased by pretreatment with C547 and that duration of the protective effect of this slow-binding AChE inhibitor could be significantly longer than with pyridostigmine.

Results presented here demonstrate that C547 at the dose of 0.01 mg/kg has a long-lasting protective action of mice against POX poisoning at the dose of 0.42 mg/kg (2xLD_50_) for 96 h, whereas pyridostigmine at a dose of 0.15 mg/kg was effective less than 24 h.

## Results

### Effect of reversible pre-inhibition of AChE with C547 on the time course of irreversible inhibition by paraoxon

**Scheme 1:**
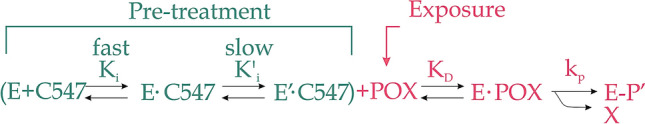
Minimum reaction scheme of POX with AChE in the presence of C547.

For initial proof of concept that slow-binding reversible AChE inhibitor can decrease the rate of AChE inhibition by OPs, we used an in vitro model. Human recombinant AChE was inhibited by various concentrations of C547 until the reversible inhibition steady state was reached, then POX was added. The concentrations of C547 were selected in accordance with previously obtained data^39^. Progressive inhibition of AChE by POX under pseudo first-order conditions in the presence of C547 can be described by Scheme 1, in which binding of C547 prevents binding and phosphorylation of AChE by POX.

**Figure 1:**
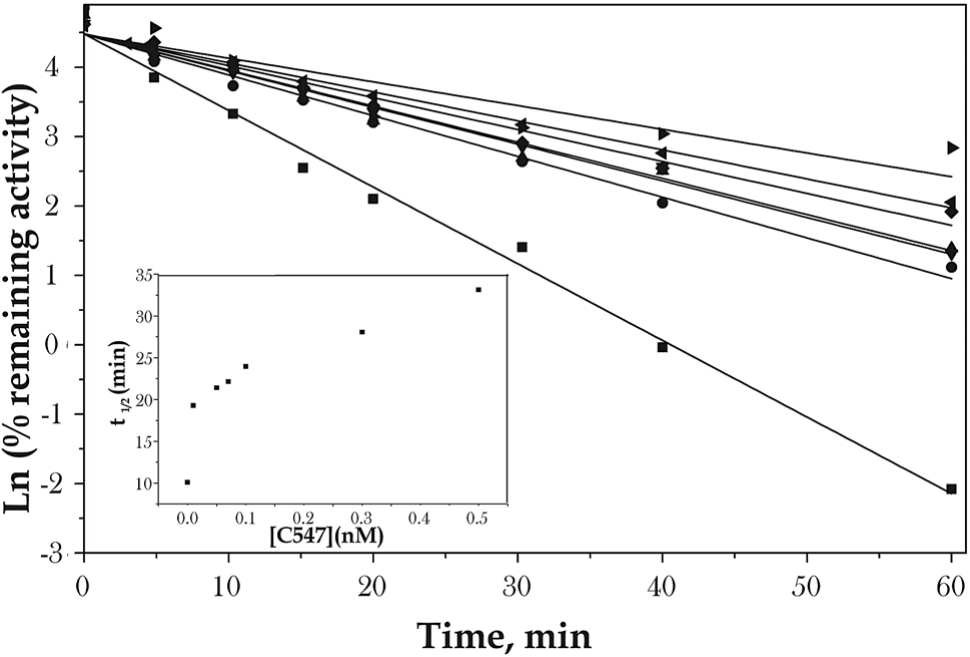
Time course of inhibition of human AChE under first order conditions by 10 nM POX after 5 min of pre-incubation with different concentrations of C547 (● 0.01 nM, ▲ 0.05 nM, ▼ 0.07 nM, ◆ 0.1 nM, ◂0.3 nM, ▸0.5 nM, ■without C547) in 0.1 M sodium phosphate buffer, pH 8.0 at 25ºC. Slope of each plot is the observed first order rate constant (k_obs_). Insert: Increase in the half-time of first-order phosphorylation process (t_1/2_ = ln2/k_obs_) of human AChE by 10 nM POX as a function of C547 concentration.

The mechanism of slow-binding of C547 to human AChE was previously investigated^39^. It is a slow-binding reversible process of type B: after formation of an initial complex at the entrance of the active site gorge, an induced-fit step leads to a tighter complex in which C547 spans from the top to the bottom of the gorge. Thus, in Scheme 1, E^.^C547 is the initial enzyme complex, E’^.^C547 the final complex, E.POX the reversible enzyme^.^POX complex, E-P’ the phosphorylated enzyme, and X, the POX leaving group, *para*-nitrophenol. The irreversible inhibition half time, t_1/2_, varied from 10.1 min in the absence of C547 to 33.1 min in the presence of increasing C547 concentration, ranging from 0.01 to 0.5 nM. The rates of inhibition of human AChE by POX after 5 min of pre-inhibition with different concentrations of C547 was found to be 1.9 to 3.2 times lower than those without C547 (Fig. 1).

### Effect of pre-treatment with C547 on paraoxon-induced muscle weakness ex vivo

As restoration of impaired diaphragm contractility appears critical for survival, we used isolated mouse phrenic nerve-diaphragm model for functional studies of the effects of C547 and POX at NMJs. It was previously shown that even at low frequency (2 Hz) of phrenic nerve stimulation POX significantly decreases the mouse diaphragm muscle contractility ex vivo^41^. It was assumed, that pre-treatment of diaphragm with C547 is able to decrease the action of POX on muscle contractions.

**Figure 2:**
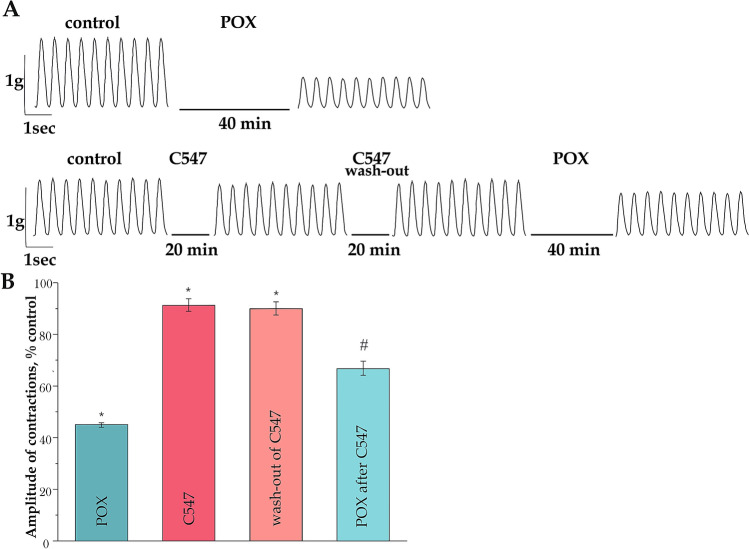
Effect of pre-treatment with C547 on paraoxon-induced muscle weakness. (**A**) Representative contractions of diaphragm recorded after exposure to 10 μM POX (top traces) and after pre-incubation in the presence of 0.1 nM C547 (bottom traces). (**B**) Relative changes in muscle contraction amplitude after inhibition of AChE by 10 μM POX. Reduction of muscle contraction force was partially prevented by pre-inhibition of AChE with C547. Amplitude of muscle contractions in control was taken as 100%. Data are expressed as mean ± SEM. *p < 0.05 compared to contractions before POX application, #p < 0.05 compared to contractions of POX treated muscles. Statistical analysis was performed using the Mann–Whitney test.

It was shown that incubating mouse diaphragm for 40 min with 10 μM POX resulted in a decrease in muscle contraction force down to 45 ± 1% of control value (p = 0.01; n = 6 muscles; Fig. 2). Pre-inhibition of AChE with 0.1 nM C547 per se also significantly decreased diaphragm muscle contraction force to 91 ± 2% of control (p = 0.02; n = 6 muscles; Fig. 2). When the steady state level of the force of contraction in the presence of C547 was reached, then C547 was washed out for 20 min using Ringer solution. It is important to note that the steady state level of contractions, reached in the presence of C547, did not change in the absence of C547 in bath solution, force of contractions was 90 ± 2% of control (Fig. 2). These results are in agreement with the contention that the slow-binding reversible AChE inhibitor is able to stay in synaptic cleft for a long time.

In the next experiments, we investigated the action of POX (10 μM) after blockade of AChE by C547 (0.1 nM) followed by excess C547 washed out. It was shown that POX exposure after 40 min pre-inhibition of AChE by C547 had a significantly smaller effect compared to effect on control muscles with no C547 pre-treatment: the muscle contraction force was reduced only to 67 ± 3% of control value (p = 0.01; n = 6 muscles; Fig. 2). Thus, ex vivo pre-inhibition of AChE with C547 is able to counteract the action of POX as irreversible AChE inhibitor on mouse diaphragm NMJs.

### Effect of pre- exposure C547 treatment on paraoxon toxicity in vivo

Since the aim of pre‐exposure treatment is to increase the efficiency of standard emergency therapy, in first instance we selected the intraperitoneal (i.p.) dose of atropine (15 mg/kg, i.p.), that administrated alone ensured the survival of about 50% of mice (Table 1), exposed to 2xLD_50_ of POX (0.42 mg/kg, i.p.). Atropine was administered one minute after challenging animals with POX. During the next sets of experiments, this atropine dose was used to study the protective properties of C547 against 2xLD_50_ of POX. C547 was administered intraperitoneally at different doses (Table 2). Then, 30 min after C547 injection, mice were challenged with POX, and atropine was administered 1 min after POX. The range of C547 doses was selected according to the effective dose previously reported for rat *myasthenia gravis* model^38^. Data are presented in Table 2. The LD_50_ dose of C547 in CD-1 mice was determined in preliminary experiments as 1 mg/kg, intraperitoneally injected.

**Table 1:** Selection of atropine dose for post-exposure therapy of mice poisoned by 2xLD_50_ of POX.

Group	n/N*
POX 0.42 mg/kg, i.p	0/24
POX + Atropine 8 mg/kg, i.p	2/24
POX + Atropine 10 mg/kg, i.p.	6/24
POX + Atropine 15 mg/kg, i.p	**11/24**

*n—number of mice survival 120 h after POX poisoning.

N—total number of mice in the group. POX – 0.42 mg/kg, i.p.

**Table 2:** Selection of C547 dose for pre‐treatment of mice poisoned by 2xLD_50_ of POX.

Group	n/N*
C547 0.005 mg/kg + POX + Atropine	12/24
C547 0.01 mg/kg + POX + Atropine	**21/24**
C547 0.015 mg/kg + POX + Atropine	18/24
C547 0.05 mg/kg + POX + Atropine	11/24

*n—number of mice survival 120 h after POX poisoning.

N—total number of mice in the group. POX – 0.42 mg/kg, i.p

C547 was i.p. administrated 30 min before POX. Atropine at the dose 15 mg/kg, i.p. was administrated 1 min after POX.

It was shown that C547 at 0.01 mg/kg has the most effective protective effect against POX. Thus, 88% of mice survived in this experimental group compared to 46% when atropine was used alone 1 min after POX exposure (Table 2). It is important to note, that there were no signs of poisoning by C547 after its administration at the dose of 0.01 mg/kg. Thereby, 0.01 mg/kg was chosen for further studies about the duration of C547 protective effect.

However, when C547 was administered at 0.05 mg/kg, signs of poisoning, i.e. muscle fasciculations were observed. Thus, C547 at a dose of 0.05 mg/kg has its own toxic side effect. This explains the decrease in animal survival (46%) compared to survival after lower doses of C547, as shown in Table 2.

In the next series of experiments, C547 was administered at 0.01 mg/kg 24, 48, 72, 96 and 120 h before 2xLD_50_ POX. Atropine at 15 mg/kg was administered within a minute after 2xLD_50_ POX. Data on the duration of C547 protective action of are shown in Table 3.

**Table 3:** Duration of C547 (0.01 mg/kg) protective action in mice poisoned with 2 LD_50_ POX.

Group	n/N*
C547 30 min before POX + Atropine	21/24
C547 24 h before POX + Atropine	22/24
C547 48 h before POX + Atropine	22/24
C547 72 h before POX + Atropine	18/24
C547 96 h before POX + Atropine	14/24
C547 120 h before POX + Atropine	11/24

*n—number of mice survival 120 h after POX poisoning.

N—total number of mice in the group. POX – 0.42 mg/kg, i.p

C547 was administrated at the dose 0.01 mg/kg, i.p. before POX. Atropine 15 mg/kg, i.p. was administrated 1 min after POX.

It was shown that C547 has a protective effect even when administered up to 4 days before POX. Moreover, the value of the protective effect of C547 administered 24 and 48 h before POX poisoning (survival was 91%) is comparable to the effect of C547 administered only 30 min before POX poisoning (88% of mice survived; Table 3). It is important to note that although POX was administered 96 h after C547, as shown in Table 3, the survival level of animals was still higher (58% of survivors) compared to the group that received only atropine (46% of survivors).

To compare duration of effects of both C547 and pyridostigmine, we selected the dose of pyridostigmine that maximizes the survival of mice pre-treated 30 min before POX injection and, then atropine (15 mg/kg) injection one minute after POX. Data are shown in Table 4.

**Table 4:** Selection of pyridostigmine dose for pre‐treatment of mice poisoned by 2xLD_50_ of POX.

Group	n/N*
Pyridostigmine 0.05 mg/kg + POX + Atropine	7/24
Pyridostigmine 0.1 mg/кг + POX + Atropine	13/24
Pyridostigmine 0.15 mg/kg + POX + Atropine	**19/24**
Pyridostigmine 0.2 mg/kg + POX + Atropine	14/24

*n—number of mice survival 120 h after POX poisoning.

N—total number of mice in the group. POX – 0.42 mg/kg, i.p

Pyridostigmine was i.p. administrated 30 min before POX. Atropine at the dose 15 mg/kg, i.p. was administrated 1 min after POX.

The most effective protective effect of pyridostigmine was observed for a dose of 0.15 mg/kg, 79% of animals in this group survived compared to 46% of surviving mice after administration of atropine alone. It should be noted that the same dose of pyridostigmine administered 24 h before POX poisoning has no protective effect. In this later group, 46% of animals survived, which corresponds to the sole effect of atropine (Table 5).

**Table 5:** Duration of pyridostigmine (0.15 mg/kg) protective action in mice poisoned with 2xLD_50_ POX.

Group	n/N*
Pyridostigmine 30 min before POX + Atropine	18/24
Pyridostigmine 24 h before POX + Atropine	11/24

*n—number of mice survival 120 h after POX poisoning.

N—total number of mice in the group. POX – 0.42 mg/kg, i.p

Pyridostigmine was administrated at the dose 0.15 mg/kg, i.p. before POX. Atropine at the dose 15 mg/kg, i.p. was administrated 1 min after POX.

**Figure 3:**
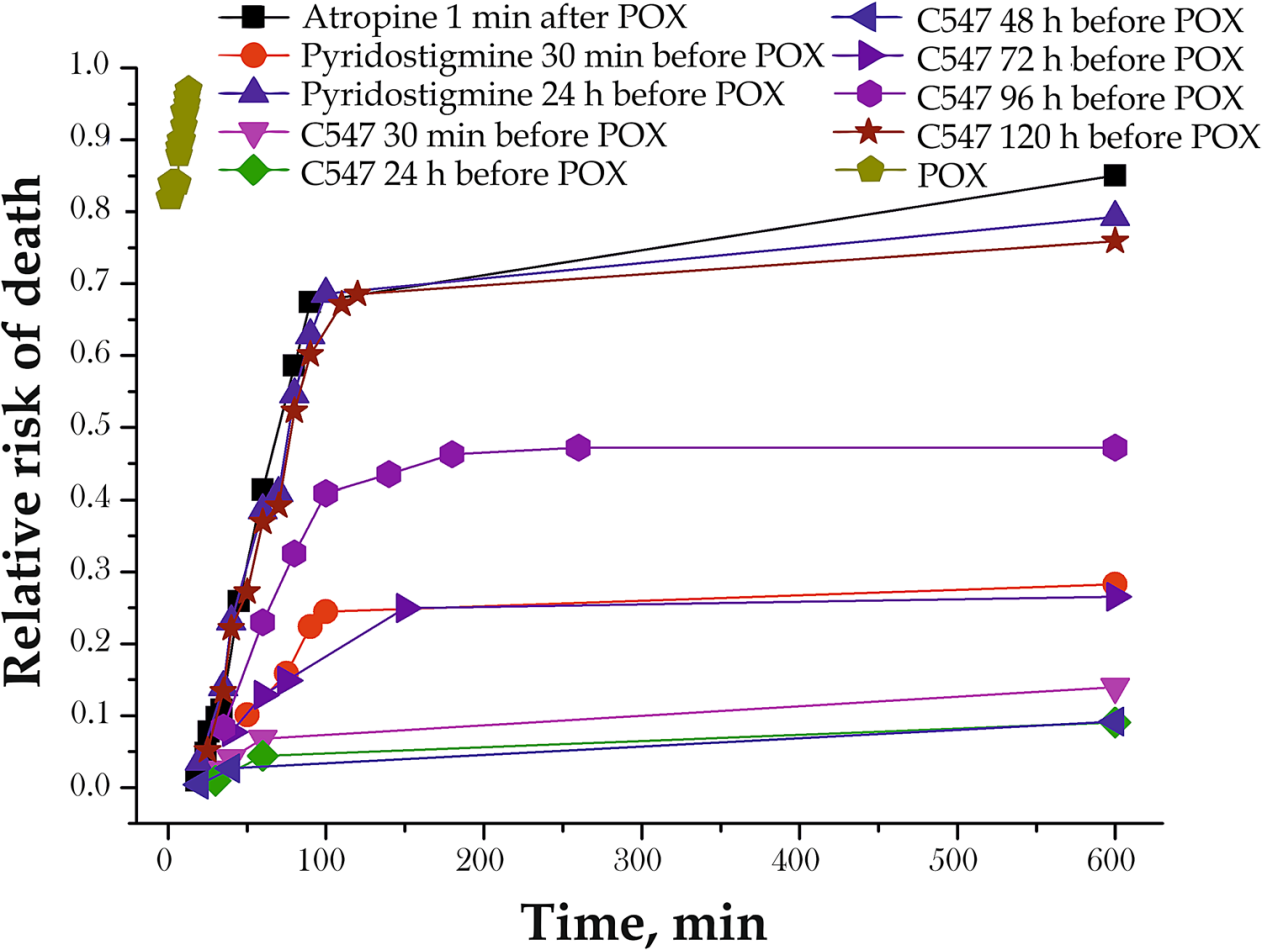
Cox analysis of survival data for mice pre-treated against POX (0.42 mg/kg, i.p) poisoning.

The relative risk (RR) of death due to POX poisoning as a function of precocity (from min to hours) of pre‐treatment by C547 or pyridostigmine was calculated according to Cox survival analysis over a period of 10 h^42^. It was shown that RR = 1 in mice exposed to POX (0.42 mg/kg). However, in animals that received atropine within 1 min after poisoning, POX-induced mortality was lower (RR = 0.7; Fig. 3).

In animals pre-treated with C547 at the dose 0.01 mg/kg, high and approximately same efficacy was observed when C547 was administered 30 min, 24 h or 48 h before POX (RR = 0.03–0.07). Increasing the time interval between administration of the same dose of C547 and POX exposure until 72 h and 96 h led to an increase in the risk of death (RR = 0.22 and RR = 0.47, respectively). Five-day (120 h) interval between administration of C547 and POX increased the level of RR to 0.7 (Fig. 3). This is the same level of RR as the group that only received atropine (Fig. 3). Thus, C547 has no protective effect at this dose if administered 5 days before exposure to POX.

In animals pre-treated with pyridostigmine (0.15 mg/kg) 30 min before exposure to POX (Fig. 3) and receiving atropine within 1 min after POX poisoning, pyridostigmine also significantly reduced the relative risk of death (RR = 0.22). However, when pyridostigmine was administered 24 h before POX exposure the relative risk of death returned to the level achieved only by immediate post-exposure atropine treatment (RR = 0.7).

Thus, C547 can be considered as a perspective compound for pre-exposure treatment of OP poisoning with a significantly longer period of protection of peripheral cholinergic system than pyridostigmine.

### Effect of C547 on locomotor and behavioral activity levels of mice

The significant caveat of toxicological experiments described here is that C547 per se, at the protective dose (0.01 mg/kg, i.p.), may induce incapacitation (physical and/or behavioral). To study whether C547 causes incapacitation, the open field test was used. This test is an in vivo assay that can detect impairment of motor as well as cognitive activities^43^. Three groups of mice (control mice and mice pre-treated either by pyridostigmine or C547) were tested before pre-treatment and 0.5, 24, 48, 72, 96, 120 h after C547 (0.01 mg/kg) pre-treatment. The locomotory (total walked distance) and exploratory (rearing, head dips) activity of mice as well as common for ChE inhibitors gastrointestinal side effects (number of fecal *boli*) were estimated.

**Figure 4:**
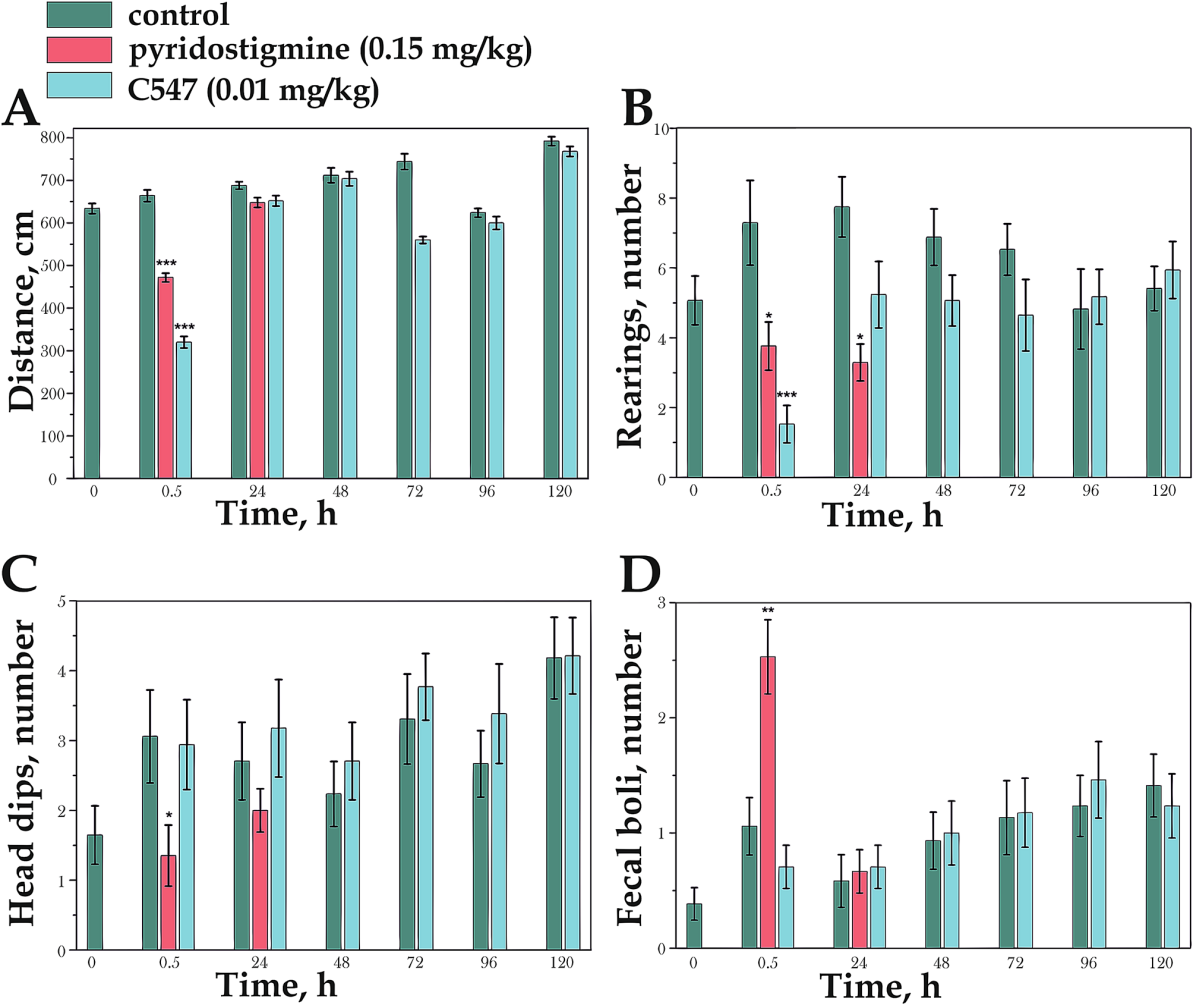
Locomotory and exploratory activity in the open field test in control group of mice, group of mice treated with 0.15 mg/kg pyridostigmine and group of mice treated with 0.01 mg/kg C547: (**A**) number of lines crossed; (**B**) number of rearing; (**C**) number of head dips; (**D**) number of fecal *boli*. Each bar represents a separate group of mice. Data are presented as mean ± SEM. *p < 0.05, **p < 0.01, ***p < 0.001 indicate significant differences by Mann–Whitney test.

It was shown that both groups of mice, treated with pyridostigmine or C547, 30 min after exposure explored the open field with significant decrease in motor activity compared to control mice in walked distance (Fig. 4A.). However, after 24 h of exposure no significant difference in walked distance with time vs. control mice was observed (Fig. 4A). Mice treated with C547 spent less number of episodes of rearing than control group after 30 min: the number of rearing decreased from 7.2 to 1.2 (p = 0.0005; n = 17 mice) (Fig. 4B). However, after 24 h there were no differences compared with control group. Pyridostigmine exposure had a lasting effect on the number of rearing up to 24 h (Fig. 4B.). There were no significant effect of C547 exposure on number of episodes of head dips, while the number of head dips of pyridostigmine treated mice was 2.4 times less than in control group after 30 min (p = 0.0004; n = 17 mice) (Fig. 4C.). Pyridostigmine at 30 min after injection increased the mean number of fecal *boli* from 1 to 2.5 (p = 0.002; n = 17 mice), while C547 had no effect on this parameter (Fig. 4D.). Thus, in open-field test, we observed inhibition of locomotory and exploratory activities in mice 30 min after exposure with pyridostigmine or C547 and recovery of activity 24 h after exposure. However, it is important to note that, unlike C547, pyridostigmine 24 h after injection also had no longer protective effect against POX.

## Discussion

One of the main objectives of this study was to establish the proof of concept that reversible AChE inhibitors for which binding kinetics (long residence time on target and re-binding) leads to long action at NMJs, can be used for prolonged pre‐exposure treatment of OP poisoning. We found that the slow-binding reversible inhibitor of AChE (C547) has a long-lasting protective action of mice against POX poisoning. It was shown that duration of C547 protective effect is much longer than the effect of pyridostigmine bromide approved for pre‐exposure treatment of OP poisoning.

**Figure 5:**
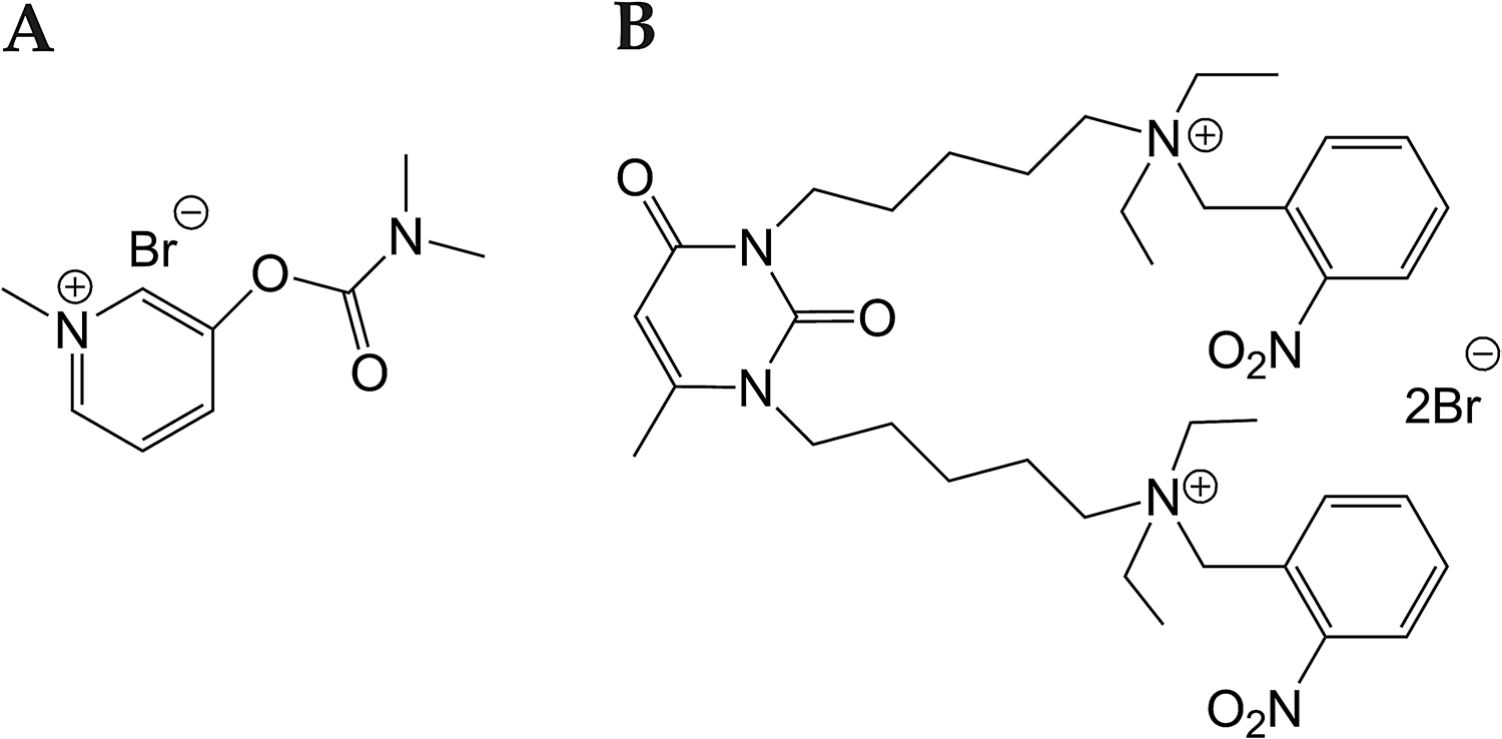
Structures of pyridostigmine (**A**) and C547 (**B**).

Pyridostigmine is the dimethylcarbamate ester of 3-hydroxy-1-methylpyridinium bromide (Fig. 5A). This carbamylating agent is not specific to AChE and also reacts with BChE^44^. BChE is of pharmacological and toxicological importance: it is an endogenous stoichiometric bioscavenger for OPs, it also metabolizes numerous carboxyl esters, including drugs and poisons^45^. Carbamates covalently bind to ChE active site serine, providing a pool of transiently inactivated enzymes, which in turn protect them against irreversible phosphylation by OP. Indeed, unlike irreversibly phosphylated AChE, carbamylated AChE slowly self-reactivates by spontaneous hydrolysis of the carbamyl adduct. For AChE inhibited with pyridostigmine, the decarbamylation time is about 70 min^46^. Pyridostigmine is rapidly eliminated from bloodstream. The elimination half-life measured after intravenous administration is 1.5 h for humans and 24 min for rats^46,47^. Therefore, duration of protective pyridostigmine effect depends mainly on life-time of carbamylated AChE, and sustained protection implies repetition of administered doses.

It has been shown that certain reversible AChE inhibitors can be used for pre‐exposure treatment of OP poisoning (for a critical review see^33^). In particular, huperzine A, administered 30 min before OP challenge, protects against 1.2 × LD_50_ soman in guinea pigs^48^ and administered up to 6 h before exposure, protects mice against 2.2 × LD_50_ soman^49^. However, there is no information that huperzine A can protect after significantly longer than 6 h period of pretreatment. Thus, further animal and PK/PD studies are needed to answer the questions whether reversible AChE inhibitors can have long-lasting protective effects, comparable to carbamates as protectants with no cognitive and physical impairments.

The main advantage of reversible inhibitors that can be used to increase the duration of action compared to carbamates is their ability to re-bind to the target. Vauquelin et al.^31^ described a micro pharmacodynamics model in which high affinity binding kinetics and long residence time play an essential role for high target occupancy, and favor rebinding to the target, when target is located in a small crowded space^29^. Therefore, it is important to stress that the unique morphological properties of NMJ may enhance the effects of AChE inhibitors. Since, NMJ functioning is directly involved in vital life processes such as respiration and locomotion, synaptic transmission at NMJ requires specific properties. The reliability of NMJ results stems mainly from the release in 3–12 times more ACh molecules than it is necessary for triggering action potential in muscle fiber^50,51^. This large excess of ACh, released under normal physiological conditions, makes this synapse extremely reliable. To terminate the action of ACh at NMJs, a very high density of AChE (5000 AChE molecules/µm^2^) is localized in the secondary folds of synaptic cleft. These morphological properties hinder diffusion of free AChE inhibitor molecules away from micro-anatomical compartment, allowing AChE inhibitors to stay at NMJs even when their concentration in the bulk phase has already dropped to very low levels. Thus, the NMJ is potentially a target‐rich compartment for the development of new therapies against acute OP poisoning.

Recently, we described the effects of the slow-binding reversible inhibitor of AChE (C547) with long-lasting action at NMJ^23^. This specific for AChE inhibitor is a derivative of 1,3-bis[5-(*o*-nitrobenzylethylammonium)pentyl]-6-methyluracilic unit (Fig. 5B). The crystal structure of mouse AChE with C547 was solved^38^. X-ray structure shows that the *o*-nitrobenzylammonium group deeply bound in the AChE gorge is stabilized by T-stacking aromatic interaction. The 6-methyluracil subpart is stabilized by aromatic stacking interactions and a hydrogen bond at the AChE peripheral anionic site. Comparison of inhibition and molecular modelling data for the non-charged related compound 1,3-bis[5-(*o*-nitrobenzylethylamino)pentyl]-6-methyluracil showed that the lack of quaternary nitrogen in the nitrobenzylammonium group abolishes both slow-binding kinetics of AChE inhibition *in vitro*^38^ and long-lasting action in vivo^35^. Thus, the bulky quaternary nitrobenzylammonium group is important for long-lasting action of C547.

The results of this study indicated that long-lasting PD of C547 due to high target occupancy and slow exit rate from micro-anatomical compartments, play important mechanistic roles to prolong in vivo action on peripheral cholinergic system of drugs, designed for pre‐exposure treatment of OP poisoning. Important to note that only 10% of residual AChE activity at the NMJ of isolated mouse hemidiaphragms exposed to POX was sufficient to allow normal muscle force generation^52^. Thus, C547 provides with pool of reversibly inhibited AChE, which protects enzyme against irreversible phosphylation by POX. However, due to reversible binding of C547, a small fraction of active enzyme is capable of working at any time, allowing ACh hydrolysis and supporting generation of respiratory muscles contractions during acute OP poisoning.

Just as is the case with pyridostigmine, due to the presence of a positive charge on the quaternary nitrogen, C547 is poorly able to penetrate the blood–brain barrier. However, it is well known that acute OP poisoning leads to death mainly due to respiratory failure caused by central apnea resulting from depression of the respiratory center and peripheral cholinergic effects, such as bronchospasm, and respiratory muscle failure^48^. The exact balance between central and peripheral mechanisms in causing death is complex and strongly dependent both on animal species and chemical nature of the OP agent. However, in humans, central respiratory failure is likely the dominant mechanism^53,54^. In addition, inhibition of CNS AChE causes seizures and irreversible brain damage.

AChE inhibitors, used in the palliative treatment of Alzheimer's disease, have been investigated for their capability to protect central AChE^16^. Thus, reversible AChE inhibitors with long residence time on target could be studied for long-lasting protection of both CNS and peripheral cholinergic system against acute OP poisoning. Thereby, our future ultimate goal is to investigate slow-binding inhibitors of AChE capable of penetrating the blood–brain barrier. Preliminary results obtained with a slow-binding non-charged transition-state analogue of ACh have encouraged us in this direction (Lenina et al., in preparation). Also, the nanotechnological approach as a strategy for delivery of C457 or similar compounds into the brain is appealing. In the last few years, nanoparticles have been successfully used for delivery of either quaternized oximes or AChE reversible charged inhibitors into the brain^55–57^. Thus, nano-cargos could be implemented to overcome the problem of the blood–brain barrier penetration for AChE inhibitors with long-lasting action as potential drugs for prolonged pre‐exposure treatment of OP-induced neurodegeneration.

## Methods

### Enzymes and chemicals

1,3-bis[5-(diethyl-*o*-nitrobenzylammonium)pentyl]-6-methyluracil dibromide (C547) was synthesized in Arbuzov Institute of Organic and Physical Chemistry, FRC Kazan Scientific Center of RAS^58^. Human recombinant AChE and POX were purchased from Sigma-Aldrich (St. Louis, MO, USA). All other chemicals were of biochemical grade.

### In vitro inhibition of AChE by paraoxon following pre-inhibition by the C547

Various concentration of C547 were mixed with human recombinant 0.5 nM AChE in 0.1 M sodium phosphate buffer (pH = 8.0). The concentration in C547 ranged between 0.01 and 0.5 nM. Thus, only a fraction of enzyme was protected by C547. The high concentration of enzyme compared to C547 concentration was on purpose so that the remaining activity after phosphorylation and subsequent dilution could be measurable. Inhibition by paraoxon (10 nM) was performed under first order conditions ([POX] > [E]). Paraoxon was added to the mixture after 5 min of incubation with C547 at 25 °C. The first order rate process of AChE phosphorylation was recorder by the classical sampling method. At various time intervals (5–60 min) aliquots (v = 20 µl) were taken and activity was measured according to the Ellman’s method^59^ in the presence of 0.1 mM DTNB and 1 mM ATC in 0.1 M sodium phosphate buffer (pH = 8.0) at 25 °C. The total volume (V) in cuvette was 2000 µl. With a volume ratio V/v = 2000 µl/20 µl, the concentration of C547 in Ellman medium dropped to 0.1–5 pM, that of POX to 0.1 nM, and that of AChE to 5 pM. Thus, because of POX concentration dropped by 2 orders of magnitude, phosphorylation immediately stopped. Also, because C547 concentration dropped much below its Ki (22 pM), the presence of C547 in the Ellman medium did not affect the measurement of residual enzyme activity.

Plots of Ln(residual activity) vs time were built. Slopes are observed first order rate constants (k_obs_). Results were expressed as t_1/2_ = Ln2/k_obs_, where k_obs_ (pseudo first-order rate constant) was calculated from the plots of Ln (% remaining activity) *vs*. incubation time with POX.

### Ex vivo twitch tension measurements

All experiments involving animals were performed in accordance with the guidelines set forth by the European Union Council Directive 2010/63/EU and the protocol of experiments approved by the Animal Care and Use Committee of Kazan Federal University. CD-1 mice weighing 25–30 g, 6-week old, were purchased from the Laboratory Animal Breeding Facility (Branch of Shemyakin-Ovchinnikov Institute of Bioorganic Chemistry, Puschino, Moscow Region, Russia) and were allowed to acclimate to their environment in vivarium for at least 1 week before experiments. Animals were kept in sawdust-lined plastic cages in a well-ventilated room at 20–22 °C in a 12-h light/dark cycle, 60−70% relative humidity and given ad libitum access to food and water.

Hemidiaphragm muscles with their associated phrenic nerves were bathed in oxygenated Ringer-Krebs’ solution at 25 °C. For twitch tension measurements the force sensor TRI201AD (AD Instruments, Sydney, Australia) was used. Contractions were evoked by stimulating the phrenic nerve via wire electrodes by supramaximal current pulses, 0.1 ms in duration. Data were recorded using Power Lab system and LabChart 6 software (AD Instruments, Sydney, Australia, https://www.adinstruments.com/products/labchart). POX and C547 were applied in Ringer-Krebs solution.

Data were expressed as mean ± SEM. Drug effect was expressed as percentage of contraction amplitude in control. Statistical significance was assessed by Mann–Whitney test at the level of p < 0.05.

### In vivo paraoxon toxicity shift assay

Stabulated animals were observed for 120 h after POX injection, and symptoms of intoxication were recorded. POX LD_50_, dose (in mg/kg) causing lethal effects in 50% of animals was determined during preliminary tests by the method of Weiss^60^. For POX toxicity shift assay at 120 h, atropine (Sigma-Aldrich, St. Louis, MO, USA) was i.p. administered 1 min after i.p. injection of 2xLD_50_ of POX. Pyridostigmine bromide or C547 were administered i.p. before POX injection. The ratio of number of mice surviving after challenge with 2xLD_50_ of POX to the total number of challenged mice was used as a criterion of toxicity shift.

### Open field test

CD1 mice were randomly divided into 3 groups (n = 17): group of mice treated with C547 (0.01 mg/kg, i.p.), group of mice treated with pyridostigmine (0.15 mg/kg, i.p.) and control group of mice. The open-field test apparatus (Open Science, Moscow, Russia) was considered of a white circular arena 62 cm in diameter with thin black lines, having 32 cm high PVC walls. The floor of the arena was subdivided into 37 parts. Behavioral assessment was carried out as described^43^. In brief, the mouse was placed in the center of lighted arena and allowed to explore for 2 min. Locomotor activity in the open field was quantified by VideoMot2 software (TSE Systems, Bad Homburg, Germany, https://www.tse-systems.com/product-details/videomot). Data were expressed as the total distance travelled in centimeters during the 2-min test. The number of rearing, head dips and defecation were considered as indexes of exploratory activity. The floor of the arena was cleaned between successive recordings with an ethanol solution (70%).

## Abbreviations

ACh: Acetylcholine
AChE: Acetylcholinesterase
C547: 1,3-Bis[5-(diethyl-o-nitrobenzylammonium)pentyl]-6-methyluracil dibromide
ChE: Cholinesterase
CNS: Central nervous system
i.p.: Intraperitoneal
NMJ: Neuromuscular junction
OP: Organophosphorus
PD: Pharmacodynamics
PK: Pharmacokinetics
POX: Paraoxon
